# Governance and implementation of the Emergency Department to Community (EDC) initiative to support complex care needs in Australia

**DOI:** 10.1108/LHS-02-2026-0026

**Published:** 2026-09-01

**Authors:** Kanchan Marcus, Philip Haywood, Gabriela Uribe, Alison Austen, Marguritte Battye, Patrick Bolton, Miranda Shaw, Shannon Saad, Paul Esplin, Althea Barry, Jenny Cox, Lisa Parcsi, Angelina Lee, Carmen Huckel Schneider

**Affiliations:** Faculty of Medicine and Health, Leeder Centre for Health Policy, Economics and Data, The University of Sydney, Sydney, Australia; Central Coast Local Health District, NSW Health, St Leonards, Australia; Murrumbidgee Local Health District, NSW Health, St Leonards, Australia; South Eastern Sydney Local Health District, NSW Health, St Leonards, Australia; Sydney Local Health District, NSW Health, St Leonards, Australia; South Eastern Sydney Local Health District, NSW Health, St Leonards, Australia; Sydney Local Health District, NSW Health, St Leonards, Australia; Central Coast Local Health District, NSW Health, St Leonards, Australia; Faculty of Medicine and Health, Leeder Centre for Health Policy, Economics and Data, The University of Sydney, Sydney, Australia

**Keywords:** Health services, Health-care delivery, Intersectoral collaboration, Evaluation, Implementation models, Australia

## Abstract

**Purpose:**

This paper aims to qualitatively evaluate the nature and strength of partnerships examining the structural factors, including information sharing and policy alignment that support the transition of patients from emergency department presentations across New South Wales, Australia.

**Design/methodology/approach:**

An evaluation using a qualitative design and analytical framework was undertaken. Documentary analysis and semi-structured interviews and focus groups were conducted with stakeholders, allied health professionals and community partners. Findings from these sources were triangulated and presented at a Roundtable to gather feedback and insights.

**Findings:**

Emergency Department to Community (EDC) implementation reflects localised approaches; in a rural local health district (LHD), the initiative is transitioning towards a business-as-usual model, while in metropolitan Sydney LHD a formally designated virtual model is used. Yet all LHDs conduct most care coordination using telehealth modes with some face-face contact when required. Governance arrangements are effective across most sites, with implementation tailored to context. Cross sector collaborations and partnerships was a core feature of this initiative, while upstream barriers persist in primary care and regional disparities.

**Originality/value:**

This study provides empirically original insights into how the EDC model of care is governed and implemented in four LHDs, with qualitative insights from key leaders at local districts, doctors, nurses, social workers, allied health professionals, hospital managers and not-for-profit providers. By addressing social determinants of health, such as housing and access to services, these models move beyond traditional service delivery to improve both health and social outcomes for those most at risk.

Populations experiencing intersecting vulnerabilities, including multimorbidity, chronic pain, disability, mental illness, homelessness and substance use, are disproportionately affected by systemic inequities in health and social care (Marmot, 2020; Gilboe and Curran, 2025). These individuals encounter fragmented service delivery, pervasive stigma and structural exclusion, which undermine access to timely, integrated and person-centred care (Eastwood *et al.*, 2019). This can be addressed through high level multidisciplinary team care support (Fu *et al.*, 2024). Upstream policies and structures, coordinated wrap around care and equity-driven approaches that recognise the complex interplay of social determinants and health needs are required to address these disparities (Eastwood *et al.*, 2019; Wodchis *et al.*, 2020). Integrated care has emerged internationally as a key strategy for improving care coordination, reducing fragmentation and addressing complex needs of people with compounded disadvantage (Valentijn *et al.*, 2013). A European project, SUSTAIN (Sustainable Tailored Integrated Care for Older People) spanning eight countries (Austria, Belgium, Estonia, Germany, Norway, Spain, the Netherlands, UK), sought to strengthen integrated health and social care for older people through preventive, person-oriented approaches (Lette *et al.*, 2020; de Bruin *et al.*, 2020). The project identified key challenges, including fragmented funding, limited provider commitment, lack of interprofessional trust and poor information sharing (de Bruin *et al.*, 2020). Key lessons from SUSTAIN underlined the value of collaborative learning cycles and stakeholder engagement for sustainable integrated care models (de Bruin *et al.*, 2020). Furthermore, Kaiser Permanente is frequently cited as an example of an integrated care model in the USA, that brings together hospitals, primary care, specialists and community services with multidisciplinary providers and shared information (Chang, 2025). These integrated care models aim to improve patient health-care outcomes by supporting earlier, coordinated care, reduce fragmentation, enhance collaboration between providers and reduce hospital costs.

##  

### Australian context

Australia comprises a mixed public and private health-care system, with universal access to health care through Medicare, a public health insurer. The Australian Department of Social Services provides assistance to vulnerable families and implemented the National Disability Insurance Scheme (NDIS), rolled out nationally in 2020. Preventive services are focused in primary and community health care, in the state of New South Wales (NSW) while 15 Local Health Districts (LHDs) deliver public hospital services across designated regions (Uribe Guajardo *et al.*, 2025). Health and social inequities persist across Australia despite these arrangements. One in 18 hospital admissions in 2021–22 were considered potentially preventable (AIHW, 2024). Within NSW, local integrated care programs have demonstrated benefits in improving care coordination and reducing fragmented delivery over the past two decades. In 2006, HealthOne was introduced which underlined multidisciplinary care based on where people live, while in 2009, community based care was more broadly included across care sectors (Skinner, 2024). More recently, the Integrated Care for People with Chronic Conditions improved care across settings (O’CALLAGHAN, 2022), while Panned Care for Better Health, a community based care navigation program for people with psychosocial and complex needs (Hartati *et al.*, 2024), was associated with reductions in emergency department (ED) presentations. Similarly, the Healthy Homes and Neighbourhoods initiative improved engagement with vulnerable families through multidisciplinary care coordination and interrupting cycles of family disadvantage (Tennant *et al.*, 2020). Despite previous programs, many people with chronic, complex and socially disadvantaged circumstances continue to experience fragmented care and recurrent ED presentations. Consistent with NSW objectives to support value based care and coordination between hospital and community, the NSW state government commenced the roll out of the Emergency Department to Community (EDC) initiative (Baird *et al.*, 2021) in 2019, aimed to provide complex care coordination and support individuals with multiple health and social care needs who frequently present to ED. The EDC initiative uses an algorithm that flags patients that have presented 10 or more times over a 12-month period to an emergency department in NSW. Additional inclusion criteria comprise persons younger than 65 years of age (with slight variances in criteria across LHDs). EDC facilitates care continuity between ED and community services through coordination; connects patients with appropriate community services; comprises multidisciplinary teams to support complex care needs across providers and attempts to reduce repeated ED presentation through follow-up support after ED discharge (Marcus *et al.*, 2026). Common features of EDC across LHD sites include multidisciplinary care coordination to reduce service fragmentation, facilitation of referrals, navigation of health and social services, follow up support and the use of virtual care coordination such as telephone and videoconferencing, supplemented by face-to face support where required. EDC is predominantly nurse-led although staffing structures differed across LHDs and include social workers and medical doctors. While the initiative was initially supported through state government funding, services are increasingly transitioning into routine models of care.

When health and social care are disconnected, individuals with multifaceted needs experience significant gaps in support (Eastwood *et al.*, 2019). There is a growing evidence base on the use of integrated models of care to reduce ED presentations (Hartati *et al.*, 2024; Eastwood *et al.*, 2019; Baird *et al.*, 2021) and how multidisciplinary integrated care can support equity in vulnerable groups (Beacon, 2015). Given, the limited qualitative evidence on the governance, implementation, sustainability and scalability of such programs, this study aims to explore the EDC initiative, which addresses local health and social inequities. Specifically, the study seeks to qualitatively evaluate the nature and strength of partnerships examining the structural factors, including information sharing and policy alignment that support the transition of patients from emergency department presentations across NSW.

## Methods

Four NSW LHDs were purposively selected to participate in the evaluation because they were among the earlier sites to implement the initiative and collectively represented diverse demographic and geographic contexts. This sampling included LHDs with differing population characteristics and urban, regional and remote profiles which enabled examination across settings which would further support transferability to the broader health system. A contextual summary of the four LHDs is provided in Table 1.

**Table 1.** Contextual summary of the four participating LHDs and the national disability insurance scheme in Australia

LHD	Description
Central Coast LHD	CCLHD is a regional area, comprising an estimated 346,596 people and a rapidly growing Aboriginal and Indigenous population at 4.9% in 2021, above the Australia average of 3.2%
Murrumbidgee LHD	MLHD is a regional and remote area, spanning 125,243 square kilometres. The population of MLHD was estimated at 249,164 persons in 2022, with majority of the median age 55–64 years in 2021 (8%–8.5%)
South Eastern Sydney LHD	SESLHD’s population will exceed one million by 2031 (10% growth). Chronic conditions are increasing: 50% overweight, 21% with multiple conditions, 16% with high psychological distress and 12% of hospital admissions preventable. In 2021, 40% of residents were overseas-born, 30% from non-English-speaking countries. Languages include Mandarin, Cantonese, Greek, Arabic, Spanish, Nepali and others. Aboriginal population reached 10,350
Sydney LHD	The number of residents within SLHD boundaries is expected to increase from over 740,000 to 819,540 by 2036, with around 1% identifying as Aboriginal or Torres Strait Islander. Nearly half the SLHD region speak a language other than English at home, with Chinese, Arabic, Greek, Korean, Italian and Vietnamese. SLHD has a high number of residents facing housing instability, and around 28,000 people live with a disability
National disability insurance scheme	The national disability insurance scheme (NDIS) is an Australian government program that provides funding support to people with disabilities. A registered provider is able to provide daily living assistance, care, therapy, transport or support into specialist accommodation. This is managed under the national disability insurance agreement

Note(s):

Australian Bureau of Statistics. 2021. Central Coast (NSW), (2021) “Census All Persons QuickStats [Online]”, Australian Bureau of Statistics, available at: Link to the website of Central Coast (NSW) (accessed 13 September 2025). Marcus, K., Haywood, P., Uribe Guajardo, M.G., Austen, A., Battye, M., Bolton, P., Shaw, M., Saad, S., Esplin, P.A., Barry, A., Cox, J., Parcsi, L., Lee, A. and Huckel Schneider, C. (2026). “Strengthening emergency department to community partnerships: qualitative insights from Four local health districts in New South Wales”, *Journal of integrated care (Brighton, England)*, Vol. 34, pp. 28–45. MLHD (2024). “MLHD at a glance; Population and Health Indicator Data 2024”. in NSW Health, Murrumbidgee LHD Public Health Unit. SESLHD (2024). “Population Profile of Priority Populations in South Eastern Sydney Local Health District”, Based on 2021 ABS Census Data. NSW Government, Sydney. SLHD. Sydney Local Health District Planning Tools [Online]. Sydney: NSW Government, available at: Link to the website of Sydney Local Health District (accessed 15 September 2025)

The research objectives were as follows:

1. to describe the operational context and policy supports of the EDC initiative across LHDs, including comparative analysis;
2. to qualitatively explore partnerships’ taxonomy (networks, communication);
3. to explore information sharing between LHDs and community-based EDC partners; and
4. to describe the processes involved in community of practice, specifically how EDC patient information is shared between LHDs.

### Data collection

A 12-month evaluation using a qualitative design was undertaken. Documentary analysis and semi-structured interviews and focus groups were conducted. Findings from these sources were triangulated and presented at a stakeholder Roundtable to gather feedback and insights.

Documents were provided from site leads at each participating LHD, to the research team with digital copies of EDC planning and implementation materials. All documents contained de-identified data related to implementation, planning and governance activities. Interviews and focus groups were conducted (online) with stakeholders, allied health professionals [general practitioners (GPs), managers, social workers, nurses] and community-based partners (not-for-profits, government departments, National Disability Insurance Scheme providers). Detailed sampling and recruitment methods are described elsewhere (Blinded). A Roundtable was held in hybrid mode, comprising managers, medical professionals, social workers, nurses, allied health professionals and academics. Participants were purposively selected and invited to the Roundtable by LHD leads based on their involvement with the EDC initiative.

### Analytic framework

The evaluation design was informed by a series of theoretical frameworks (Kang, 2016; Lewis *et al.*, 2008; Wodchis *et al.*, 2020). Kang’s Intersectional Collaboration Framework provided insight into levels of collaboration, including communication capacity, staffing, governance structures and funding mechanisms. Lewis *et al.*’s Network Structure Dynamics and Sustainability Framework guided the classification of partnerships, encompassing elements such as information sharing, participation, organisation, management and support. As data collation progressed, Wodchis Policy Support Framework emerged as the most relevant, and was subsequently adapted to better reflect the policy and supports central to the evaluation’s focus (Wodchis *et al.*, 2020). Analysis was undertaken thematically grounded within Strauss and Corbin methods (Corbin and Strauss, 2008).

### Ethics approval

Ethics and site approvals were obtained (Blinded). All participants provided informed consent prior to taking part in the study.

### Data analysis

There were 48 documents and 53 participants from interviews and focus groups across the four LHDs. Implementation and planning documents comprised community of practice, strategic plans, templates, governance structures, multidisciplinary team meeting frequency and resourcing needs. Demographic details for each document were initially extracted for; title, date (if available); author, format of document, purpose, methods/results, limitations. Sub-themes were identified through repeated back and forth inductive and deductive processes. The same process of analysis was undertaken for all interviews and focus group transcripts. After both forms of data analysis were complete, the sub-themes were triangulated into an adapted version of Wodchis framework (Wodchis *et al.*, 2020) and key themes identified.

## Findings

The numbers of patients enrolled into the EDC program each month by each LHD varies between 15 and 20 and up to 50 patients (SESLHD). See Table 2 for EDC operational models and Table 3 for the comparative analysis of consent, enrolment and discharge processes by LHD. Findings are presented under two major themes; EDC systems and processes and Beyond boundaries: integrated care and social partnerships. The following sections present participant accounts and associated sub-themes with representation from all four LHDs and a range of professional roles.

**Table 2.** EDC operational models in four LHDs

CCLHD	MLHD	SESLHD	SLHD
Commenced EDC in 2022 Nurse led model Integrated care team leads EDC alongside PCBH initiatives Criteria >15 presentations in ED Most care coordination is virtual via phone/video Sometimes patient is EDC discharged and put onto the Integrated Chronic Care list for face-face consultations and long-term care MoH funded a project role for 12 months Activity Based Funding is the measure for services	Commenced EDC in 2021 Nurse led model Integrated care team leads EDC alongside PCBH initiatives Criteria >10 presentations in ED Most care coordination is virtual via phone/video. For this cohort mostly face to face Shifting model to Interprofessional Practise Model to business as usual MoH funded a project role for 2 years Activity Based Funding is the measure for services	Commenced EDC in 2019 Nurse-Social Work led model with access to Medical Officer EDC sits under Population and Community Health Criteria >17 presentations in ED Hybrid – face to face and virtual via phone/video 4x FTE staff for EDC; nurses and social workers Activity Based Funding is the measure for services	Commenced EDC in 2022 Medical-Social Work led model EDC sits within RPA Virtual Hospital Criteria >10 presentations in ED Virtual model with 24/7 access – majority of coordination is via phone/video Face to face offered if required RPA Virtual Hospital is block funded to deliver a range of virtual models of care MoH funded a project role for 12 months 2021-2022: $100K transitional funding at commencement for EDC and Planned Care for Better Health initiatives Activity Based Funding is the measure for services

**Source(s):** Authors’ own work

**Table 3.** Comparative analysis of consent, enrolment and discharge processes by LHD

LHD	EDC Patient consent process	EDC Enrolment process	EDC Discharge process
*CCLHD*	Implied consent for internal staff NSW Health After a deep file review, individual cases brought to the Multi-Disciplinary Team meeting (MDT) meeting At MDT; case is reviewed and if yes to intake then care coordination with internal providers and GP without direct patient consent into initiative Attempt to gain consent from clients with complex issues to liaise with external services e.g. NDIS and community service providers	EDC list in Patient Flow Portal Deep dive file review of each individual case Gaps identified in patient care Each case is brought to the MDT MDTs and ED Management Plan and/or Ambulance plan if appropriate Care coordination mostly by phone/email/ SMS/video EDC list in Patient Flow Portal or direct referral from elsewhere	Care coordination supports are in place; NDIS paperwork/provider in place NDIS service providers given strategies to prevent client from having an avoidable hospital presentation Discharged to Drug & Alcohol, Mental Health, Integrated chronic care team, Aboriginal health or other specialities Ensure access to their community GP Patient not responding to calls after multiple attempts, ‘Unable to contact letter’; occasionally F-F visit
*MLHD*	Implied consent for internal staff NSW Health Consent obtained for external coordination with NGOs, NDIS and community providers, Police, Corrections, Patient to join MDT	EDC list in Patient Flow Portal Interprofessional Practise Model Capacity building all staff to support patient centred care with multidisciplinary team Complex cases brought to an Inter-Professional Practise Forum for shared care planning MDTs and ED Management Plan Care coordination mostly by phone/email/F-F Important to engage with the consumer where they felt most comfortable	Care coordination supports are in place Drug & Alcohol, Mental Health or other specialities’ capacity building to lead case Discharged to Marathon Health/NGO Linked into community GP
*SESLHD*	Implied consent for internal staff NSW Health Consent obtained for external coordination with NGOs, Department of Communities and Justice and community providers	EDC list in Patient Flow Portal Gaps identified in patient care Each case is brought to the MDT ED Management Plan Care coordination by phone and f-f; home visits; café, locating person in park	Care coordination supports are in place Housing support through DCJ/NGOs NDIS coordination/supports/ guardianship Drug & Alcohol, Mental Health taken lead Centrelink support with paperwork Proactively engage patients not ready to accept community supports
*SLHD*	Implied consent for internal staff NSW Health Direct ED referral: consent obtained in ED (new process since 2025) At the MDT; case is reviewed and if yes to intake then the patient is contacted for consent into EDC initiative	EDC list in Patient Flow Portal Gaps identified in patient care Deep dive file review of each individual case Each case is brought to the MDT ED Management Plan Care coordination mostly virtual (some f-f if required)	Care coordination supports are in place; violence support, bereavement services, short term mental health support Homelessness support through DCJ Drug & Alcohol team have taken lead Linked into community GP

**Source(s):** Authors’ own work

### Emergency Department to Community systems and processes

Participants reported that the target population served by the EDC initiative presents with highly complex care needs, including socioeconomic disadvantage, co-occurring health and social challenges, mental health issues, substance use, disability, chronic conditions and housing instability:

[…] majority of people are significantly impacted by the social determinants of health […] (MLHD, Allied Health Professional).

### Governance processes and operations

Program implementation began in 2019 for SESLHD, in 2021 for MLHD and in 2022 for SLHD and CCLHD. Early implementers of the program – the SESLHD and MLHD – were instrumental in sharing key learnings with other LHDs, as the complexity of the cohort and coordination became evident in enrolment numbers:

[…] they embedded it into their existing offering and leveraged off existing staff and relationships and care pathways, but what became really clear was the ED to community cohort essentially the frequent users of ED were a highly complex group of people […] needed social work, needed NDIS, potentially aged care, needed so much inter-agency police, guardianship – it was complex and that took years to develop that kind of relationship […] so we had a community of practise, we had lots of learnings, we were sharing and we were trying to support but it was challenging furthermore, and that very much reflected in enrolment numbers […]. (MLHD, Stakeholder).

[…] I think we may have been the first ED to community team across the state and then since that time we’ve seen it grow across other local health […] also we’ve helped other teams over time with their experience […] (SESLHD, Allied Health Professional).

CCLHD was “launched” into the EDC initiative with minimal funding support yet demonstrated resilience by successfully integrating the program into existing workflows:

[…] was the only way that we were able to sustain it on the Central Coast was a challenge initially […] and we had to look at how do we transition and implement that into our business as usual model so we did look at aligning with existing services and built that into their current role. (CCLHD, Stakeholder).

The EDC mode of care sits organisationally within the virtual hospital in SLHD, with access to nurses 24/7 (Wilson *et al.*, 2025). Face-to-face coordination occurs more so in SESLHD (where staffing allows) and is provided in CCLHD, MLHD and SLHD when necessary.

Most significantly, the majority of participants across LHDs consistently described Multi-Disciplinary Team (MDT) meetings or Case conferences as essential and highly effective in fostering cross-sector collaboration. Localised MDT meetings were reported to be well-structured in most sites, supported by tools like Microsoft Teams:

[…] it’s taken many, many years to actually build rapport with some of our internal stakeholders […] trying to break down those silos like instead of going oh, that’s not our job […] one of the successes there for us […] our CE recognised that as one of the goals so we have that executive buy-in and it will then become other people’s business to actually you know they had KPIs attached to it so they were probably more willing to engage and could see the benefit […] (MLHD, Allied Health Professional).

Community-based participants suggested the initiative could be more strategically embedded at an executive level within organisational structures. They considered the success of this model hinges on the support of key individuals who bridge hospital services with the community, highlighting the significant work to sustain collaboration across sectors:

[…] continuing those agreements with organisations like with us […] those accountability meshes just in terms of making sure that the meetings do happen […] 'cause everyone’s really under the pump and we’re all under resourced – It’s a really hard time – I actually think that senior executives – it needs to be a directive that this needs to happen and we need to work with these organisations because people aren’t and there needs to be much more opportunity to collaborate with us in the Community to breakdown these silos […] [non-government organisations (NGO), MLHD].

### Referral, enrolment criteria, consent and discharge

Participants echoed that the types of patients enrolling in the EDC initiative often present with highly complex trauma. EDC serves to shift care from high-cost settings to more cost-effective community-based locations (Wilson *et al.*, 2025), while still addressing the intensive needs of patients with complex trauma histories.

The state Ministry of Health implemented a “Patient Flow Portal” to streamline identification and enrolment into the EDC initiative. This system proactively flags potential patients based on predefined criteria, enabling timely engagement. In addition, supplementary referral pathways from allied and specialist services ensure comprehensive coverage. In 2025, SLHD began direct Ed. referral pathways to streamline care and improve efficiency. Proactive referrals from NSW Ambulance were similarly undertaken in CCLHD and MLHD. Ambulance collaboration was seen positively by participants, supporting early and preventive oriented care through shared care plans and reduced ED presentations.

Stakeholders, allied health staff and community-based participants reported a desire to refer more clients into the EDC initiative; however, referral and enrolment criteria were unclear to ED staff. Specifically, it would be beneficial for criteria guidance:

[…] If you’re trying to refer somebody out of hours to a community team, well, there’s no phone number you can call at 2:00 in the morning, where do you fax the form […] from an emergency point of view, we’re dealing with the issue in front of us today and we see 300 patients a day […] we need to know the clear criteria we need to know exactly what they were targeting and we’d have to know what’s the type of patients you’d want for that programme […] and if it’s too hard, we’re not going to do it realistically […] (SLHD, Stakeholder).

In other instances, participants also reported informal referral pathways through meetings and networks. Capacity constraints were also stated by participants across all four LHDs:

[…] we have a meeting at Sutherland quite regularly with the two staff specialists that do the ED management plan – they’ll talk about clients that may not be at our 17, but they do use a lot of staff resources in the ED so we will have a look at them […] (SESLHD, Allied Health Professional).

Allied health participants reported confusion regarding the consent process given that privacy requirements are barriers to interagency cooperation. Many EDC patients have behavioural issues and NDIS carers need to be contacted to obtain the full health profile and history:

[…] it’s quite challenging for coordinated care that we can’t actually speak with anyone outside of the LHD […] because a lot of EDC clients that I’ve had, they’re a lot of drug and alcohol issues, a lot of mental health issues, a lot of social issues […] the model of care could be worked on for a little bit more guidance around you know what exactly is expected. (CCLHD, FG, Allied Health Professional).

Discharge processes included liaising across community sectors, allied professions, primary care GPs, psychologist services, housing, NGOs and other LHDs.

### Beyond boundaries: integrated care and social partnerships

#### Cross border collaborations.

Participants reported that cross-border LHD meetings were helping to overcome longstanding fragmentation of care across health districts, enabling sharing of patient information and plans and more coordinated support for patients who move between hospital boundaries. While challenges remain in determining lead responsibility, social workers and informal pathways have proven essential in facilitating collaboration. Notably, strengthened partnerships between LHDs have led to more integrated care pathways:

[…] we’ve got the statewide clinical hub that we’ve set up for different LHDs that comes together so the clinical hub is was set up by Sydney and Northern Sydney, so us and Northern Sydney, it brings together all the different LHDs that have presenters who present across LHD boundaries […] it’s been really valuable […] we get shared ED management plans […] (SLHD, Allied Health Professional).

### Primary care service providers and GP accessibility

Participants agreed that EDC patients face major barriers to GP access, driven by high costs, limited availability and reduced bulk-billing (a government subsidised payment under Medicare). Patients with complex needs struggle to find GPs who can provide comprehensive care, as funding models undercompensate time and resources, making it financially unattractive for GPs. Information sharing between hospital to primary care remains outdated, with some providers still relying on faxes in 2025:

[…] no bulk billing GPs and unless they do it out of the goodness of their heart, or I do all the work and then […] send the information to them and they look at it after hours or they send it back to me, but they can’t even sit in on an MDT […] they want people to not get sick and they want ED avoidance but we don’t have the things in there to give them the ED avoidance. (MLHD, Allied Health Professional).

Participants valued the work that GPs do and empathise with their workload:

GPs are awesome and they do a great job, but then they have a lot of responsibilities. (CCLHD, Allied Health Professional).

Barriers in mental health triage were reported by participants which prevent individuals in lower priority categories from accessing the support they need:

[…] if it goes to mental health, they need to have the criteria to meet mental health case management and that’s quite a high criteria […] I would say at the moment 90% of my clients have borderline personality disorder so that does not come into mental health it just makes it more complex with trying to get anything done […] they’ve fallen through the gaps […] (SESLHD, Allied Health Professional).

### Not-for-profit community providers and NDIS

In a rural LHD, strong ties with Marathon Health – a non-government partner, enabled EDC implementation through a skilled workforce and flexible, trust-based collaboration, including selected information sharing practices. The After-Hours Crisis Referral Service also linked hospitals with NDIS providers, while NGOs played a vital role in care coordination and cross-sector partnerships with social services and GPs. Community participants voiced potential to scale and replicate these partnerships with similar organisations to enhance hospital-community integration in LHDs:

[…] persistence and consistency and really they need to learn to trust you […] a little bit more favourably than another NGO that may be seen as being maybe less credentialed […] we’re accountable we deliver […] we’ve had to earn our relationship with the LHD over time […] our staff are really passionate […] if we all work together, we’re going to reduce lengths of stay, reduce hospitalizations […] there’s training opportunities, we offer it to each other like we can go in and do it together that helps embed […] (NGO, MLHD).

Community providers offer the unique advantage of seeing patients in their own environments, enabling a more holistic understanding of their circumstances – an insight that hospital or GP settings may not fully capture:

[…] one of our things from our organisation is quality, accessible healthcare regardless of where you happen to or choose to live, which means that we put our staff say like in integrated care, we’ve got about 15 staff […] they’re embedded in their local community so we do a risk assessment […] we will have to request to meet them in a public space, or we need to find a second staff member as well […] key thing is building a relationship […] we’ll get a referral from their GP and it’s like living fine, et cetera and we go there and they’re sleeping in their car because their house is completely hoarding and they haven’t actually slept in their house for years, so it creates a picture of what we can then feedback […] (NGO, MLHD).

NDIS providers played a varied role in care coordination, with skill levels and responsiveness differing across the sector. While some providers were effective and valued partners, others struggled, particularly when clients faced complex needs such as mental health issues or behavioural challenges. Training or education support was suggested by allied health professionals:

[…] NDIS is really helpful because they do the after-hours where they’re doing the home visits, but sometimes they’re perhaps would benefit from more education around health, physical health, comorbidity, drug health, mental health, those sorts of things to build their capacity around how to respond […] I think that would be really helpful so providing training and education and perhaps, involving them in some of the meetings that we have in health as well […] I think they’d appreciate being included in that […] building those relationships and you know like using interpersonal skills and communication skills […] (SLHD, Allied Health Professional).

Ongoing challenges persist between the NDIS and the hospital sector, particularly around unclear expectations regarding roles, responsibilities and information sharing barriers. Challenges were also stated for the interface between mental health and disability sectors:

[…] some clients who are on NDIS getting them to come into a facility when a patient is admitted for social reasons really because they are homeless and their mental health and she’s an indigenous woman […] so then NDIS wouldn’t come in because we were a hospital and we should be doing everything for her […] (MLHD, Allied Health Professional).

### Housing and justice system

Housing support for vulnerable individuals was reported to involve coordinated efforts across multiple organisations, including NGOs, NDIS providers, health staff and GPs working together to streamline care. However, in rural areas, challenges arise when individuals are housed in locations lacking access to specialist health services or equipment, highlighting an opportunity to strengthen integrated planning across sectors and regions.

Ongoing challenges remain around determining whether Health, Department of Communities and Justice (DCJ) or NGOs should take lead responsibility, alongside difficulties in securing long-term mental health support for individuals with complex needs:

[…] we will get clients housed, but in order to sustain this tenancy, they need ongoing mental health support […] they’re housed, and there might be a couple of approaches where community mental health has tried to see the client – have not managed to see the client and have withdrawn […] we need them to stay involved on a weekly basis, checking in, engaging […] helping people manage their mental health […] it’s not something that we see with any consistency or regularity […] (DCJ, Community Provider).

Some LHDs voiced strong relationships with the NSW housing department. Community-based participants also stated successful examples of coordination with housing:

[…] we’ve got a really good service we can provide the support they need in terms of housing and linking in with Homes NSW. Our team has a very good relationship with Housing and through that we can often try and advocate strongly and get people housed maybe a bit quicker […] (SESLHD, NGO).

However, housing individuals with complex behavioural and mental health needs presents significant challenges, particularly when legal and policy frameworks intersect.

### Rural and remote contexts

Rural and regional areas face chronic under-resourcing. Participants stated that patients moving from urban to remote areas created major barriers to their care, with limited local services leading to ED admissions and telehealth consultations seen as insufficient. Coordination was further hindered by disconnects between metropolitan-based providers like NDIS:

[…] we use a lot of what’s called the remote medical service and the patients after hours […] they have a virtual consult, and often the result of the virtual consult will be admit for the night and let your doctor sort it out in the morning, which is not really conducive to what we’re trying to do with these people and people then tend to know that if I present to the hospital after 8:00 at night, I’ll get a bed Yeah. (MLHD, Allied Health Professional).

[…] the ones that probably work the least successfully are if I’ve got a person in Cootamundra and their NDIS provider or support plan coordinators’ in Sydney […] there’s so much remote […] support care coordination is massive and it’s really hard ‘cause they’re not here – they really need to develop a relationship and a rapport with people, and if you can’t do that, especially with the really complex consumers, there’s no trust and they’ve been in and out of the system […] (MLHD, Allied Health Professional).

Roles that are unfilled remains a challenge and some participants stated the desire for more permanent based roles:

[…] we’ve got Aboriginal healthcare workers who are great, but there’s positions empty at the moment […] it’s just not filled […] and we’ve had a high number of tragic deaths and suicide in one of our higher Indigenous population towns […] Permanent roles, not just projects anymore ‘cause they can’t roll on to anybody like we literally are doing more with less […] (MLHD, Allied Health Professional).

## Discussion

Findings underline that the EDC targets a vulnerable cohort, particularly impacted by social determinants of health, including trauma. This aligns with the review on *Health Equity in England: The Marmot Review 10 Years On,* where underlying causes of ill health are interlinked to social circumstances (Marmot, 2020). In our study, localised context, infrastructure and population needs appropriately shaped EDC implementation. Cross sector collaborations and strengthening partnerships with primary care GPs, not – for-profits, housing, justice and NDIS providers was a core feature of this initiative. Governance and operations followed a broadly similar structure, highlighting the critical role of communication within and across organisational sectors, leveraging online tools, multidisciplinary team meetings and face-to-face connections, alongside community-based discharge coordination with NGOs and NDIS providers. Telehealth emerged as a consistent enabler across local contexts, while participants emphasised the need for strategic embedding at executive levels to sustain resources and reinforce the program’s role in supporting the most disadvantaged. Upstream barriers for transitioning to primary care persist including information sharing barriers between hospital to community, GP accessibility, mental health service gaps and rural and remote challenges. NGOs and NDIS providers bridged some of these health and social care gaps, though provider skill varied and while housing supports were beneficial, long-term tenancy remained problematic, especially for highly vulnerable individuals with disability, behavioural, mental health and justice-policy complexities. Despite structural constraints, integrated care models like EDC play a vital role in breaking down silos and supporting society’s most vulnerable populations.

Operational models relied upon telehealth for collaboration and communication, supplemented by face-to-face consultations when needed. A systematic review of 24 studies on chronic conditions found that the quality of care outcomes between a telehealth consult and in-person care was not different, and fewer ED visits occurred after telehealth encounters (Sina *et al.*, 2025). While evidence supports the benefits of telehealth consultations in remote areas with adequate digital infrastructure, a qualitative study involving health staff highlighted the continued importance of face-face interactions (Mathew *et al.*, 2023), particularly for Aboriginal peoples, non-English speaking communities and individuals with low literacy.

Shared governance decisions around EDC enrolment and discharge were made during multidisciplinary meetings, led by allied health staff. However clearer criteria are needed for other hospital and community teams including the emergency department staff. Multidisciplinary collaboration underlines the fundamental aspect of trust building and power sharing involved in integrated care models, which aligns with prior research (Eastwood *et al.*, 2019). Integrated care requires holistic support that moves beyond traditional health-care silos to meet individualised needs of patients, which is also echoed in a qualitative study in the USA on prenatal care coordination (Gillespie *et al.*, 2025). The EDC initiative combined targeted case management with structured care coordination, where staff took ownership of guiding patient journeys of an extremely vulnerable cohort and building strong networks with service providers, allied health professionals and specialists. These findings further coincide with the Checkpoint study (Baird *et al.*, 2021) and the Planned Care for Better Health initiative which showed reductions in unplanned ED hospitalisations as a result of timely care coordination, interdisciplinary collaboration and the importance of coordinators in bridging care gaps (Hartati *et al.*, 2024).

Infrastructure for exchanging patient information across hospital and primary sectors is acknowledged in the evidence base (Trankle *et al.*, 2019; Wodchis *et al.*, 2020). Ongoing effort is needed to maintain cross-sectoral collaboration, with informal communication and relationship building which have been critical enablers to cross-LHD meetings. While collaboration with community providers has improved, upstream challenges persist in workforce gaps in regional areas and mid-level factors such as educating health-care providers about available services and referral pathways. Despite limited resources, NGOs continue to play a key brokering role in filling health and social care gaps, especially in rural settings. Gilboe and Curran echo similar findings in health justice partnerships, with a need for joined up collaborations, rights-based approaches and emphasis on bottom-up grassroots actors that help address social determinants (Gilboe and Curran, 2025).

System level barriers in primary care, such as out-of-pocket costs and limited access to GPs or psychologists pose significant equity challenges. Consistent with other studies, GPs reported spending enormous time coordinating care for vulnerable patients and expressed a need for more integrated support (Fu *et al.*, 2024). Upstream technological infrastructure would be required for information sharing not only across hospitals (underway in NSW through one digital patient record, 2026) but also into community settings. Our study also identified high mental health triage thresholds and limited availability of long-term psychological support. A concentration index analysis of better access mental health services revealed persistent inequities, with disadvantaged and remote areas experiencing lower service volumes and less access to specialist care (Meadows *et al.*, 2015). Additionally, capacity building for NDIS providers to manage complex needs was found. These changes require upstream, policy reforms in primary care and resources to support NDIS provider skills.

A strength of this study is that it involved consultation, engagement and feedback with a steering committee comprising representatives across the urban and regional districts. Qualitative insights reached data saturation which means that no new insights were reported after 20 interviews. A further strength was the inclusion of a diverse range of stakeholders, allied health professionals and community-based partners. A key limitation is that the collection of patient reported experiences was not feasible within the 12-month design – this should be a vital focus in future research. Of note, findings about resources will be described in a separate paper.

### Implications for health and social care initiatives

This Australian study of the EDC initiative exemplifies strengthening collaboration and partnerships across organisations and sectors and reducing health and social care silos to support the most vulnerable. This approach aligns with state policy objectives to decrease acute presentations and strengthen preventive care through integrated care teams. Empowering localised health-care teams with resources to support programs that are relevant to population specific needs is vital in reducing inequities and sharing power. For example, in a remote LHD, a not-for-profit was filling essential service gaps where vulnerable individuals were “falling through the cracks”. Public policy and resource allocation should prioritise reducing health inequities through integrated, patient-centred solutions that operate across multiple levels – policy, organisational and community sectors. This requires fostering cross-departmental and cross-sector partnerships to address upstream social determinants of health, including housing, education and employment alongside clinical care. Initiatives like EDC exemplify how inclusive, team-based models can catalyse systemic change by navigating structural power dynamics and improving equity in access to services.

## Conclusion

The EDC initiative addresses structural barriers and supports joined up collaborative care for underserved populations, and those experiencing compounded disadvantage. EDC governance and implementation is undertaken with multidisciplinary teams that includes health professionals, clinicians and community representatives, which is locally adapted to meet infrastructure and population needs. Virtual communication underpins care coordination, supported by strong cross-sector partnerships. Despite systemic challenges in primary care and persistent regional inequities, innovative, capacity-building approaches are extending care to vulnerable populations in community settings. By addressing social determinants of health, such as housing and access to services, these models move beyond traditional service delivery to improve both health and social outcomes for those most at risk.

## Data Availability

The data that support the findings of this study are not available for sharing due to privacy and ethical restrictions. All findings are included in the published article.

## Artificial Intelligence (AI)

All content created is based from study findings as interpreted by authors and feedback from the broader team. AI was not used.
